# Native T1 mapping allows for the accurate detection of the segments with chronic myocardial infarction in patients with known or suspected coronary artery disease

**DOI:** 10.1186/1532-429X-18-S1-P70

**Published:** 2016-01-27

**Authors:** Yoshitaka Goto, Masaki Ishida, Akimasa Yamada, Mio Uno, Shiro Nakamori, Motonori Nagata, Yasutaka Ichikawa, Kakuya Kitagawa, Masaaki Ito, Hajime Sakuma

**Affiliations:** 1 grid.412075.5Radiology, Mie University Hospital, Tsu, Mie, Japan; 2 grid.412075.5Cardiology, Mie University Hospital, Tsu, Mie, Japan

## Background

Late gadolinium enhancement (LGE) cardiac magnetic resonance (CMR) is the current gold standard for the assessment of the myocardial infarction (MI). However, LGE CMR requires the administration of gadolinium contrast medium, which is contraindicated in patients with advanced chronic kidney disease (CKD). As patients with advanced CKD have higher prevalence of MI, accurate non-contrast CMR technique to detect MI is valuable. A previous study showed that visual assessment of native T1 map at 1.5T provided high specificity but only moderate sensitivity for detecting chronic MI. However, diagnosis performance of current native T1 mapping at 3.0T for detecting chronic MI has not fully understood. The aim of this study was to investigate the diagnostic accuracy of segmental native myocardial T1 mapping for detection of MI in patients with known or suspected CAD.

## Methods

Consecutive 30 patients with known or suspected CAD who underwent CMR at 3.0T including cine, native T1 mapping and LGE were studied. Patients with acute MI, coronary artery bypass surgery and non-ischemic cardiomyopathy were excluded. T1 mapping was performed with a modified Look-Locker inversion recovery (MOLLI) sequence on 3 left ventricular (LV) short-axis slices (basal, mid, and apical). Endo- and epicardial borders of the LV myocardium were manually traced on each slice. Myocardium was divided into AHA 16 segments. Segmental mean T1 values were quantified with a heart rate correction. On the corresponding slices of LGE images, presence or absence and transmural extent of MI were visually determined in each segment by consensus of two experienced observers. Presence or absence of severe wall motion abnormality (severe hypokinesis to dyskinesis) was also determined in each segment on the corresponding slices of cine images.

## Results

MI was present in 10 of 30 patients (33.3%) in LGE CMR. Of the 480 segments, 475 segments were eligible for the analysis after excluding 5 segments due to artifacts on MOLLI images. MI was seen in 40 segments (8.4%) on LGE images. In 13 of the 40 segments, the transmural extent of MI was >50%. Native T1 was significantly greater in the segments with MI than those without MI (1491.5 ± 122.2 ms vs 1335.1 ± 95.9 ms, p < 0.001). The area under the ROC curve (AUC) of native T1 for detecting the segments with any MI was 0.891, providing sensitivity and specificity of 75.0% and 88.9%, with a cut-off of 1420 ms. For detecting the segments with MI with transmural extent of >50%, AUC of native T1 was 0.925 and the sensitivity and specificity was 92.3% and 86.5%, with the same cut-off. No apparent wall motion abnormality suggestive of MI was observed on cine MRI in 25 of 40 segments with MI. Native T1 mapping accurately detected the presence of MI in 17 of the 25 segment (68%).

## Conclusions

The current results demonstrated that native T1 mapping at 3.0T allows for the accurate detection of the segment with MI in patient with known of suspected CAD without administrating gadolinium contrast medium.

**Figure 1:**
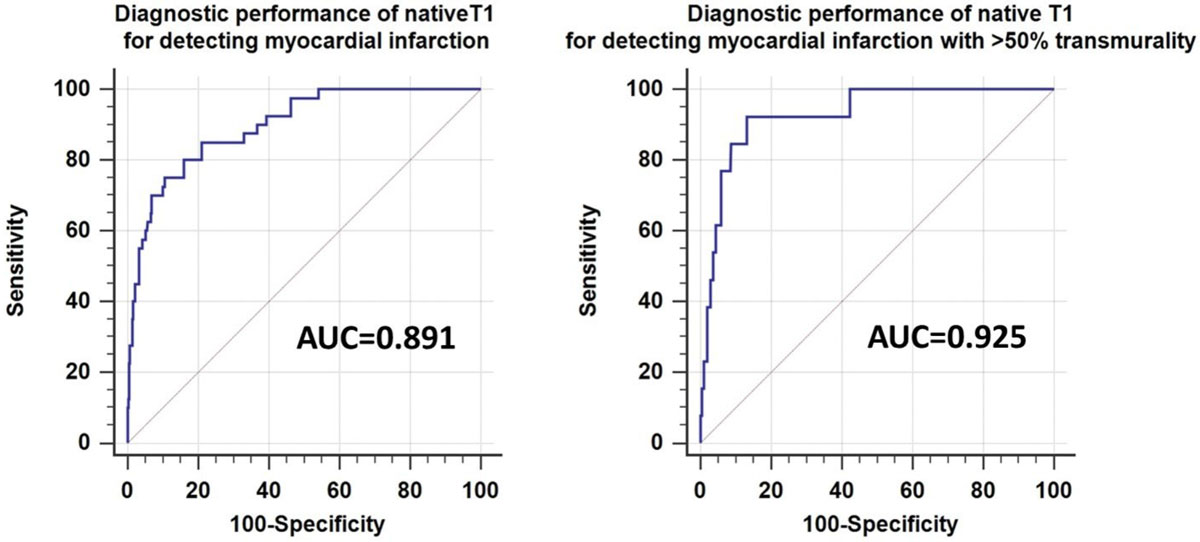
Figure 1

